# Patient-specific and hyper-realistic phantom for an intubation simulator with a replaceable difficult airway of a toddler using 3D printing

**DOI:** 10.1038/s41598-020-67575-5

**Published:** 2020-06-30

**Authors:** Junhyeok Ock, Eunseo Gwon, Doo-hwan Kim, Sung-hoon Kim, Namkug Kim

**Affiliations:** 1 Department of Convergence Medicine, Asan Medical Institute of Convergence Science and Technology, University of Ulsan College of Medicine, Asan Medical Center, 388-1 Pungnap2-dong, Songpa-gu, Seoul, South Korea; 2 Department of Radiology, University of Ulsan College of Medicine, Asan Medical Center, 388-1 Pungnap2-dong, Songpa-gu, Seoul, South Korea; 3 Department of Convergence Medicine, Asan Medical Institute of Convergence Science and Technology, University of Ulsan College of Medicine, Asan Medical Center, 88 Olympic-Ro 43-Gil Songpa-Gu, Seoul, South Korea; 4 Department of Anesthesiology and Pain Medicine, University of Ulsan College of Medicine, Asan Medical Center, 88 Olympic-Ro 43-Gil Songpa-Gu, Seoul, South Korea

**Keywords:** Anatomy, Engineering, Diagnosis

## Abstract

Difficult tracheal intubation is the third most common respiratory-related adverse co-morbid episode and can lead to death or brain damage. Since difficult tracheal intubation is less frequent, trainees have fewer opportunities to perform difficult tracheal intubation; this leads to the need to practice with a hyper-realistic intubation simulator. However, conventional simulators are expensive, relatively stiffer than the human airway, and have a lack of diversity in terms of disease variations and anatomic reproducibility. Therefore, we proposed the development of a patient-specific and hyper-realistic difficult tracheal intubation simulator using three-dimensional printing technology and silicone moulding and to test the feasibility of patient-specific and hyper-realistic difficult intubation simulation using 3D phantom for the trainee. This difficult tracheal intubation phantom can provide a realistic simulation experience of managing various difficult tracheal intubation cases to trainees, which could minimise unexpected tissue damage before anaesthesia. To achieve a more realistic simulation, a patient-specific phantom was fabricated to mimic human tissue with realistic mouth opening and accurate difficult airway shape. This has great potential for the medical education and training field.

## Introduction

There are several reasons for the presence of difficult tracheal intubation, including facial deformity, acromegaly, an airway tumour, and diabetes^1,2^. Difficult tracheal intubation without proper airway management is the third most common episode of respiratory failure and can lead to hypoxemia, possible brain damage, or even death related to difficult with ventilation^2^. One of the most important skills of trainees is difficult tracheal intubation^3^; however, patients with difficult tracheal intubation are less frequent, and trainees have fewer opportunities to practice difficult tracheal intubation skills in these patients^4^. In addition, there is limited clinical data on the difficult tracheal intubation of toddlers^5^. Therefore, practicing difficult tracheal intubation management through simulators is an appropriate alternative and has been recommended in many previous studies^3,4^. However, high-fidelity simulators are expensive^3,6^, and most simulators are stiff and less flexible than human tissue^4^. In addition, there is a lack of variety of disease models and descriptions of human anatomy. Medical applications using three-dimensional (3D) printing technology are rapidly spreading and used in a variety of ways including patient-specific implants^1,7^, simulators for rehearsal surgery^1,8^, phantoms for patient education^2^, and image-based surgical guides to display accurate resection areas^1^. Considering the high price of the simulator and several additional limitations, there have been studies to develop a low-cost difficult tracheal intubation simulator using 3D printing^3,6^. In addition, a previous study has compared conventional simulators with 3D printing and animal model simulators^9^. However, existing studies have only evaluated the airway using 3D printing attached to a conventional simulator^1,3,6,9^.

Therefore, we proposed the development of a patient-specific and hyper-realistic difficult tracheal intubation simulator using 3D printing and silicone moulding based on a patient’s medical images, and by considering anatomic movements such as those of the jaw and cervical spine(c-spine) as well as tongue pressure. We also test the feasibility of a difficult intubation simulation using the tracheal intubation phantom for the trainee.

## Results

### Fabrication of intubation simulator

**Figure 1:**
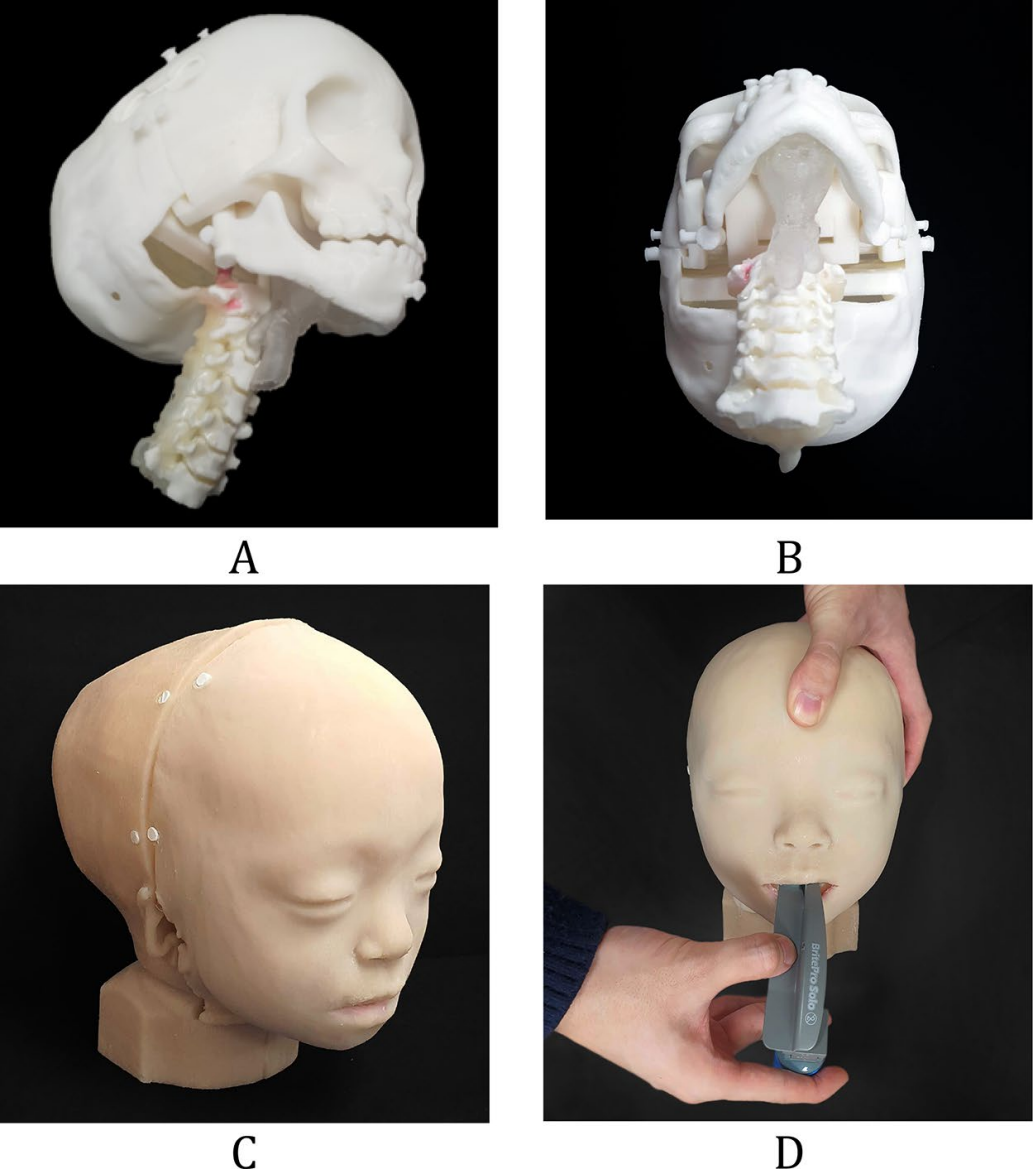
A patient-specific and hyper-realistic phantom for difficult tracheal intubation simulator. (**A**) Assembly of the inner parts, including the back of the skull, cranio-maxilla, mandible, cervical-spine, airway, and tongue in the isometric view. (**B**) At state, (**A**) bottom view. (**C**) Final phantom with front and rear skins. (**D**) Intubation with Macintosh blades in the phantom with mouth opening.

Crouzon syndrome is a genetic disorder characterized by craniosynostosis which means premature fusion of skull bones. Many features of Crouzon syndrome result from the premature fusion of the skull bones, including wide-set bulging eyes, strabismus, a beaked nose, underdeveloped upper jaw, and dental problems. Some Crouzon syndrome patients have an opening in the lip and the roof of the mouth, so-called cleft lip and palate^10^. In clinical Practice, meticulous management for these patients is of importance because these airway anomalies can cause difficult intubation and are prone to be complicated with respiratory problems. We fabricated each anatomic part using various 3D printing technology with different materials, which were subsequently assembled into one phantom. Figure 1 depicts the assembly of the inner parts, including the back of the skull, cranio-maxilla, mandible, c-spine, airway, and tongue, and assembly of the front and rear skins of a patient-specific and hyper-realistic phantom for a difficult tracheal intubation simulator. Intubation with Macintosh blades was tested on the phantom by mouth opening. In addition, the cost of silicone moulding and 3D printing of the phantom was $270 without considering labour costs. In addition, silicone moulding would need some experience. However, if there are proper models, 3D printing of phantoms needs less expertise.

### Shape accuracies

**Figure 2:**
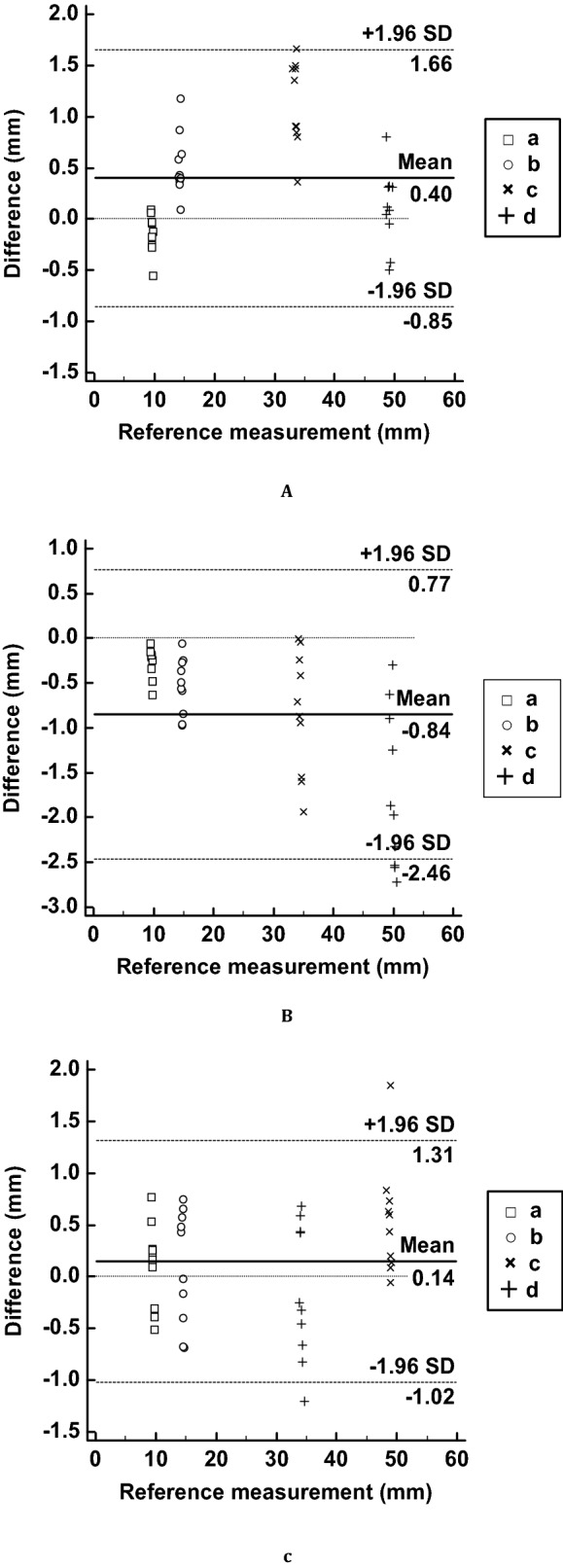
Bland–Altman plots to evaluate the accuracies between the STL file and the three airway and tongue parts with different 3D-printing methods. (**A**) STL vs Objet J750 with agilus, (**B**) STL vs Form2 with elastic, and (**C**) STL vs X-fab with flexa 693. The lengths were measured at (a) the diameter of the airway, (b) width of the epiglottis, (c) length of at the epiglottis to the end of the airway, and (d) length of the tongue.

The most important element to express in the difficult tracheal intubation simulator is the airway and tongue parts. We fabricated three airway and tongue parts using different 3D printing machines, including Form2, X-fab, and Objet J750. Two researchers independently measured four lengths five times to evaluate shape accuracy. The measurements of the STL and the 3D printed airway and tongue parts were performed and evaluated using the Bland–Altman plot (Fig. 2). The measurement error [mean ± standard deviation (SD)] of the parts by Objet J750 with agilus, Form2 with elastic, and X-fab with flexa 693 was 0.40 ± 0.58 mm (limit of agreement from − 0.85 to 1.66 mm), − 0.84 ± 0.77 mm (limit of agreement from − 2.46 to 0.77 mm), and 0.14 ± 0.58 mm (limit of consensus from − 1.22 to 1.31 mm), respectively. All the measurements, except for the distance of the length of the tongue (Fig. 7A[d]), were within the 95% confidence interval. Kruskal–Wallis test was used to analyse the difference between the airway and tongue parts and STL. The average rankings for the Objet J750 with agilus, Form2 with elastic, and X-fab with flexa 693 were 54.84, 69.00, and 57.66, respectively, and the P-value was 0.156.

#### Hardness of the airway and tongue

**Figure 3:**
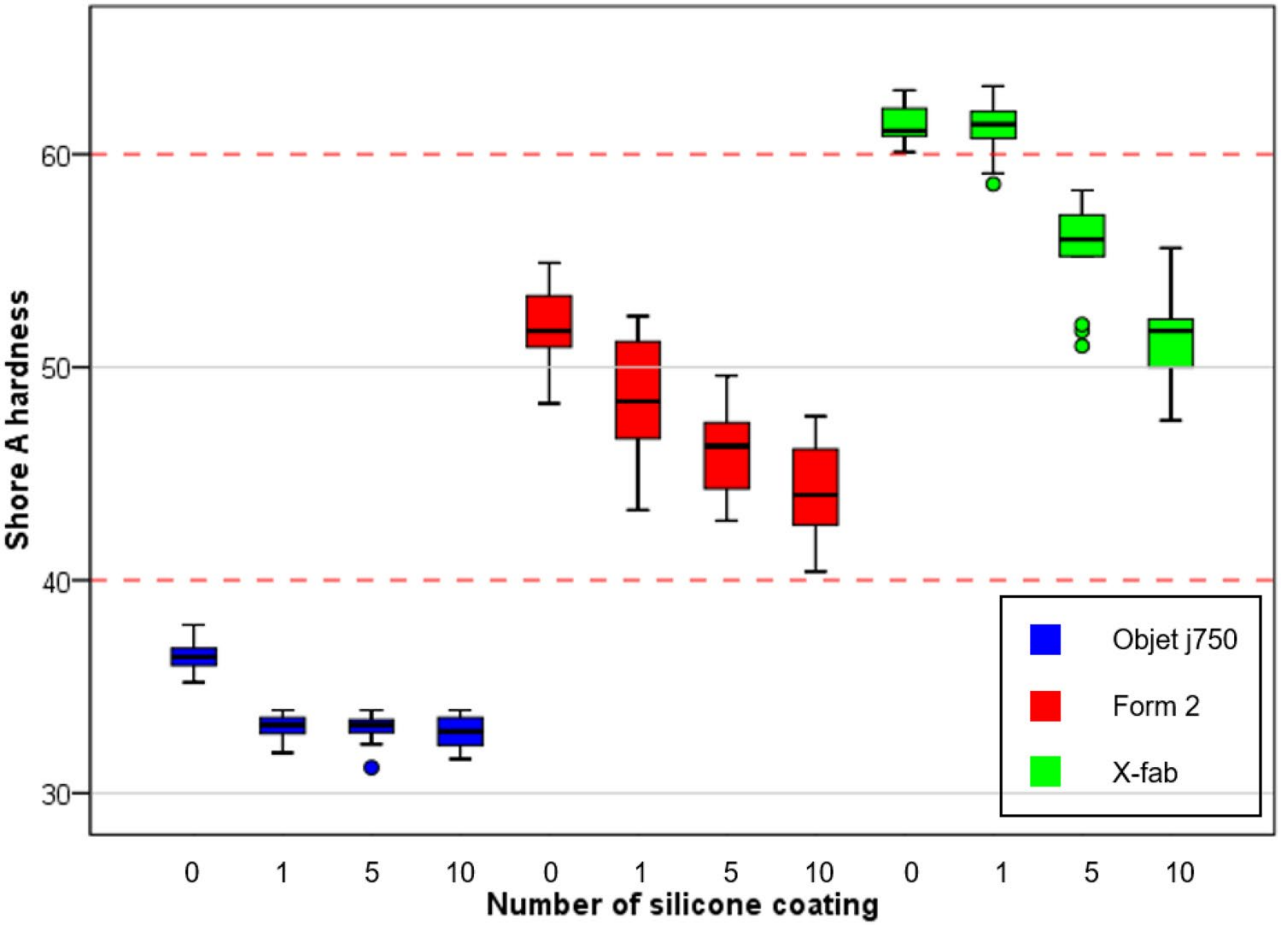
Box plot displaying measurements of Shore A hardness of printing methods including Objet j750 (blue), Form2 (red), and X-fab (green) with the number of Med6-6606 coatings. Specimens with 0, 1, 5, and 10 coatings. The box represents the middle 50% of the data and the quartile (IQ) range. The whiskers represent the maximum and minimum values less than 1.5 times the IQ range.

We fabricated three airway and tongue parts using Form2, X-fab, and Objet J750. To measure the hardness of the airway and tongue parts, specimens of 30 × 30 × 10 mm were fabricated with different numbers of silicone coatings. Five measurements at the edge and centre of three specimens per condition were performed using a Shore A durometer (Fig. 3). The hardness decreased as the number of coatings increased. The measurements (mean ± SD) of specimens fabricated by Form2 with MED6-6606 coating of 0, 1, 5, and ten times were 51.92 ± 1.99 A, 48.52 ± 3.04 A, 46.05 ± 2.16 A, and 44.22 ± 2.32 A, respectively. The measurements of X-fab were 61.39 ± 0.91 A, 61.24 ± 1.27 A, 55.70 ± 2.35 A, and 51.56 ± 2.29 A, respectively. The measurements of Objet j750 were 36.47 ± 0.75 A, 33.13 ± 0.57 A, 33.04 ± 0.65 A, and 32.85 ± 0.79 A, respectively. A previous study demonstrated that the Shore A hardness of newborns and children was between 40 and 60 A^9^. Among these tests, specimens by Form2 had Shore A hardness among the reference hardness with 0–10 times coating. Specimens by X-fab had below 60 A hardness in cases with over five coatings. In cases of Objet j750, Shore A hardness was below 40A for all specimens.

#### Accuracy of mouth opening

**Figure 4:**
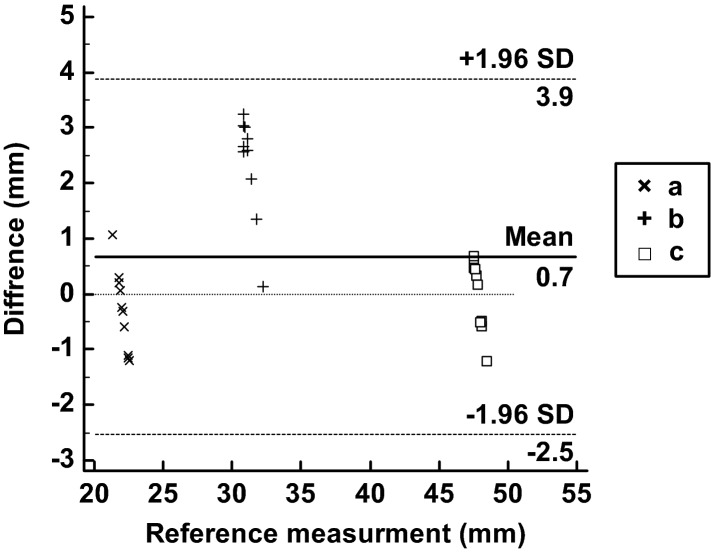
Bland–Altman plot to evaluate differences between the STL file and the phantom. (a) Measurements of STL and printed phantom at a 21 mm inter-incisor distance, (b) 32 mm inter-incisor distance, and (c) 47 mm inter-incisor distance.

One of the most important factors of this phantom is the accuracy of mouth opening. The opening distance of the inter-incisor was set to 21, 32, and 47 mm. Two researchers measured these distances five times to analyse the accuracy of the opening distance of the mouth. The measurements of the STL and the printed phantom were evaluated using the Bland–Altman plot. The measurement error (mean ± SD) was 0.67 ± 1.42 mm (limits of agreement from − 2.5 to 3.9 mm) (Fig. 4). All the measurements were within the 95% limits of agreement.

## Discussion

We have demonstrated that the hyper-realistic difficult tracheal intubation phantom has physical properties that enable functional task alignment during patient-specific training for difficult tracheal intubation in a toddler. The phantom was similar in appearance to patients with Crouzon syndrome. The mouth opening, tongue displacement secondary to laryngoscopy blade insertion, and stiffness during head extension were similar to those experienced when intubating real patients as assessed by experts. The airway and tongue parts were fabricated by three different 3D printing machines, including Objet j750, Form2, and X-fab, to evaluate the shape accuracy and to mimic the mechanical properties of the human airway and tongue. Using a Bland–Altman plot, X-fab, and Objet j750 we showed the reasonable errors (limit of agreement, 1.22 to 1.31 mm, and − 0.85 to 1.66 mm, respectively). Form2 fabricates a somewhat smaller size with a relatively large error (limit of agreement, − 2.46 to 0.77 mm). With regard to mechanical properties, the hardness of the airway and tongue parts were evaluated and compared with those in the study by Al-Ramahi et al. in which the Shore A hardness of newborns and children was estimated to be between 40 and 60 A^9^. The hardness of the specimens fabricated by Form2 with five or more silicone coatings were within this range, while those by Objet j750 were outside of the target range. However, the softness of Objet j750 could be used to fabricate realistic airways with various mechanical properties to mimic the human airway and tongue parts. The opening distance of the mouth was measured using the inter-incisor distance, and the measurement error between the STL and the printed phantom showed reasonable accuracy (limits of agreement, − 2.5 to 3.9 mm).

In previous studies, we evaluated the patient characteristics and anatomical landmarks of the upper airway in young children based on MRI images to facilitate more optimised management of oropharyngeal or nasopharyngeal airways^10,11^. Allied to limitations in anaesthesia training time assignments, the drive to improve quality and safety in health care has strongly encouraged the development of simulators for training^12^. Successful simulation-based learning is achieved by aligning functional tasks within an appropriate context whereby pre-existing skills and educational needs are matched to simulator complexity and task difficulty^13^. In this respect, manufacturing a 3D prototype is widely useful for industrial purposes. In the medical field, 3D models have been used in orthopaedics and neurosurgery and to evaluate heart valves^14^ and customized ergonomic laryngoscope support grips were developed to improve endotracheal intubation by using 3D printing technology^15^. With regard to airway management, models have helped to manage an adult with relapsing polychondritis^16^, to create a model that assists with the planning of single-lung ventilation^17^, or a stent for use in a child^18^. Nonetheless, most previous low-fidelity models do not provide a realistic basis for training. Difficult intubation simulation training should allow mistakes and provide as many repeatable exercises as necessary to gain competence. To achieve this, simulator interaction should mimic clinical scenarios sufficiently and appear realistic to suspend disbelief. Thus, we aimed to manufacture anatomically accurate airway simulation phantoms which are clinically very important but expected to be rarely encountered in real practice. The printed bone, articulation, and soft tissue were moulded in tissue-mimicking materials that were selected based on feedback from objective and subjective material testing. We believe this phantom is very useful to educate novice trainees on the intubation procedure for patients with a craniofacial anomaly or young children who are developmentally compromised. Future work will focus on the generation of a variety of craniofacial anomaly models with the addition of an interchangeable model design that would bring this project one step closer to imitating reality, thereby enhancing the quality of training with models.

This study has some limitations. Firstly, we only used one patient with Crouzon syndrome with difficult tracheal intubation. Follow-up studies will be conducted to fabricate various types of patients with difficult tracheal intubation phantom for more realistic applications. With regards to shape measurement, 3-matics and Vernier callipers were used to measure the accuracy of the STL model and 3D printed parts, respectively. However, the fabricated airway and tongue parts had a low hardness. Therefore, these parts were deformed when the size was measured using the Vernier callipers, which could lead to a measurement error. In future studies, phantoms should be measured by scanning the surface using a 3D scanner or CT. In addition, airway and tongue parts with varying hardness were fabricated by three different 3D printing machines, including object j750, form2, and X-fab, with varying numbers of silicone coatings. However, in some conditions, these parts could be easier to tear and possess less elasticity and stiffness than human tissues. Since 3D printing technology and new materials are rapidly developing, new printing methods and materials should be tested in future studies. Lastly, the mechanism of jaw movement could be used for various kinds of dental simulations. Although traditional phantoms designed by normal patients are commonly used to teach intubation for novice trainees, they are not able to reflect anatomic variations and related scenario of difficult intubation. We asked ten anesthesiology staff (5 trainees, 5 faculty) about reality to actual patient, usefulness to enhance comprehension, and willingness to repeated practice using current phantom as a ten-point numeric rate score. They responded 8.5, 9.1, and 7.8 average points, respectively. Now we are preparing the next study in which manufacture more various kinds of difficult intubation models such as Pierre Robin sequence, Treacher Collins syndrome, Goldenhar’s Syndrome, and Hemifacial Microsomia. We believe a more detailed classification of difficult tracheal intubation models and interactive feedback programs between instructors and trainees will enhance educational effectiveness and eventually increase patient safety.

Our next step would be to translate our phantom into the simulation process by using it as an educational tool for the teaching of difficult tracheal intubations in trainee anaesthesiologists. Educational validation will require us to define the educational process, assessment of competency, and learning outcomes. Unlike the traditional clinical apprentice model, a stable, high-fidelity simulator should offer the opportunity to provide measurable, consistent training by standardising the teaching, learning, and assessment. Questionnaire feedback can be used to determine how the phantom enhanced the trainee experiences.

In conclusion, this difficult tracheal intubation phantom could provide realistic simulation experience for trainees to manage various difficult tracheal intubation cases, which could minimise unexpected tissue damage before anaesthesia. We believe that the current 3D printing technology can narrow the gap between simulation-based medical education and authentic patient experience. For more realistic simulation, the patient-specific phantom was fabricated to mimic human tissue, with a realistic mouth opening and an accurate shape of the difficult airway, which has a great potential for the medical education and training field.

## Methods

**Figure 5:**
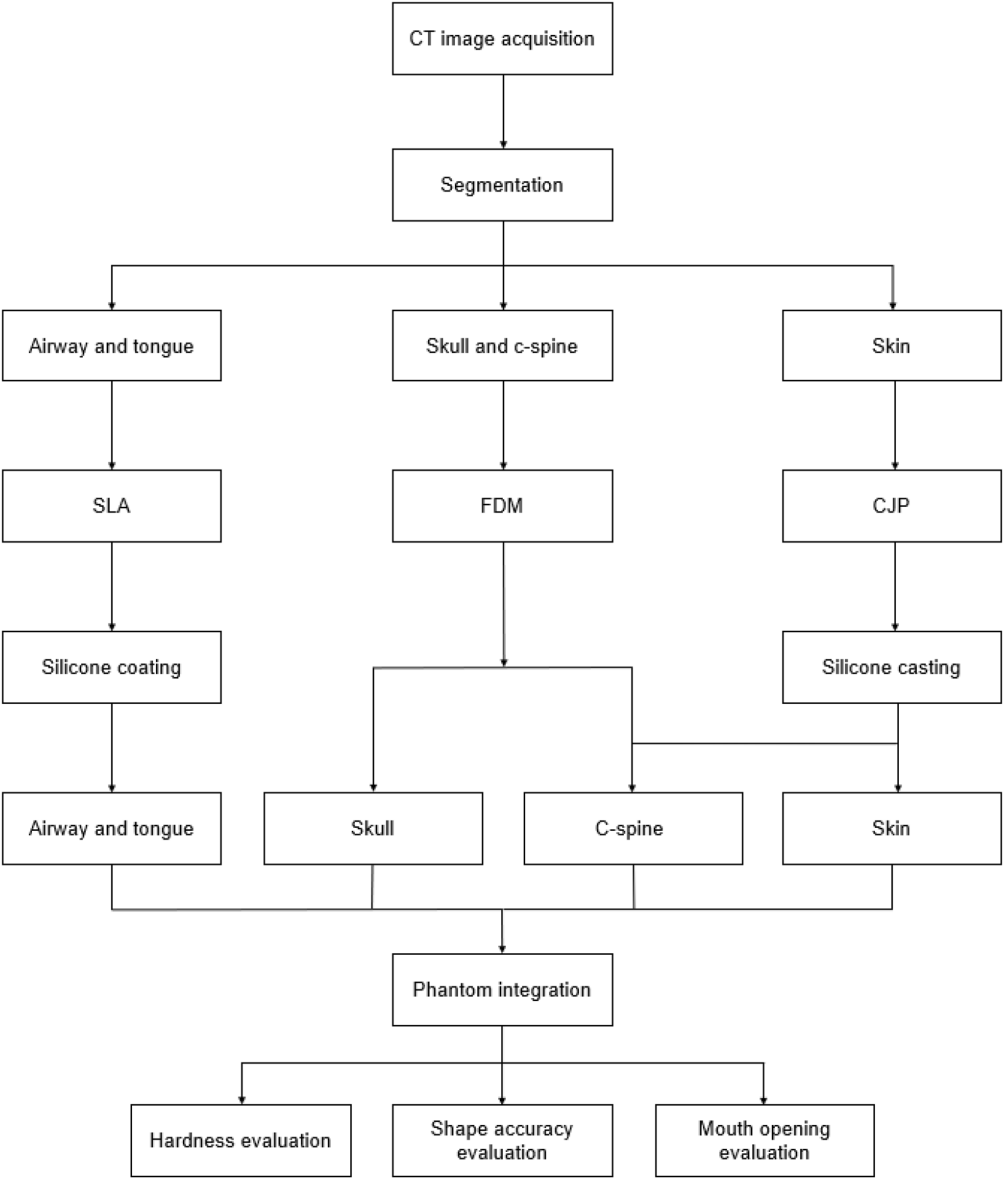
Overall procedure of manufacturing a difficult tracheal intubation simulator of a toddler. *FDM* fused deposition modelling, *CJP* colour-jet printing, *SLA* stereolithography apparatus, *C-spine* cervical spine.

3D printing technology is particularly well suited to the fabrication of various kinds of patient-specific phantoms based on medical images. To develop a more realistic phantom, 3D printing, as well as silicone casting and coating are needed. Therefore, various steps were required to fabricate patient-specific phantoms using 3D printing technology and silicone moulding simultaneously in this study (Fig. 5). First, based on medical images such as CT and MR, the segmentation of various kinds of anatomic parts involving difficult tracheal intubation such as skin, mandible, cranio-maxilla, back of the skull, airway, cervical spine (c-spine), and tongue was performed. The model was designed to consider realistic movements of the jaw and c-spine, and similar mechanical properties of the tongue, airway, and skin. In addition, the airway part was designed to be replaceable. To fabricate each anatomy part, adequate 3D printing technology and materials were chosen by considering colour, size, weight, and texture^7^. A previous study demonstrated that Shore A hardness of newborns and children was estimated to be between 40 and 60 A^9^. The simulator was evaluated by measuring the distance of the mouth opening and the shape accuracies between STL and each 3D printed phantom. In addition, the Shore A hardness of the airway and tongue was measured to assess the similarities to those of a toddler. The overall procedure of manufacturing a difficult tracheal intubation simulator is illustrated in Fig. 5.

### CT image acquisition and pre-processing

**Figure 6:**
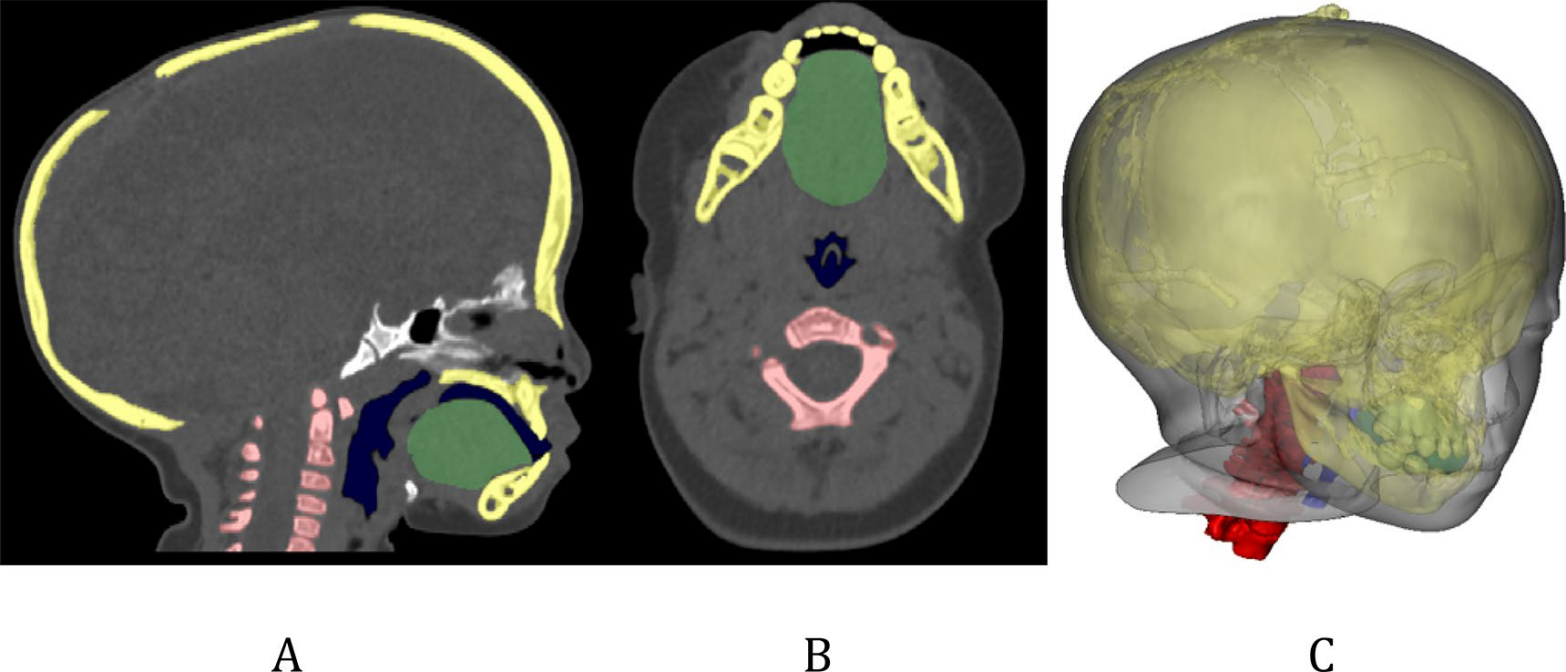
Visualisation of segmentation with various anatomic regions for designing the difficult tracheal intubation phantom in CT images of an 18-month-old patient with Crouzon syndrome. (**A**) Sagittal view, (**B**) axial view, and (**C**) 3D visualisation (cervical-spine, red; airway, dark blue; tongue, green; skull and mandible, yellow).

Head CT images were taken of an 18-month-old female patient with Crouzon syndrome who underwent Multiple detector computed tomography (MDCT) scan (Discovery CT750 HD, GE MEDICAL SYSTEMS). The CT was scanned with a 100 kVp tube voltage and a 1.25 mm slice thickness. Key parts of the difficult tracheal intubation simulator including the skin, mandible, cranio-maxilla, skull, airway, c-spine, and tongue from the CT images were segmented using Mimics v17, the medical image processing software (Materialise Inc., Leuven, Belgium) (Fig. 6). The skull and c-spine were segmented using the thresholding function (226–3,071 HU) and the region growing from manually chosen seeds by an expert. To consider the mandible movement, the skull was manually divided into the cranio-maxilla, mandible, and back of the skull. The airway was segmented using the region's growing and thresholding functions (− 1,024 to − 350 HU). The tongue was segmented with the thresholding (− 13 to 3,071 HU) function and the upper area of the hyoid bone was manually corrected. A mandible region from the skull was separated by manual delineation.

### Modelling for each anatomy

#### Airway and tongue

**Figure 7:**
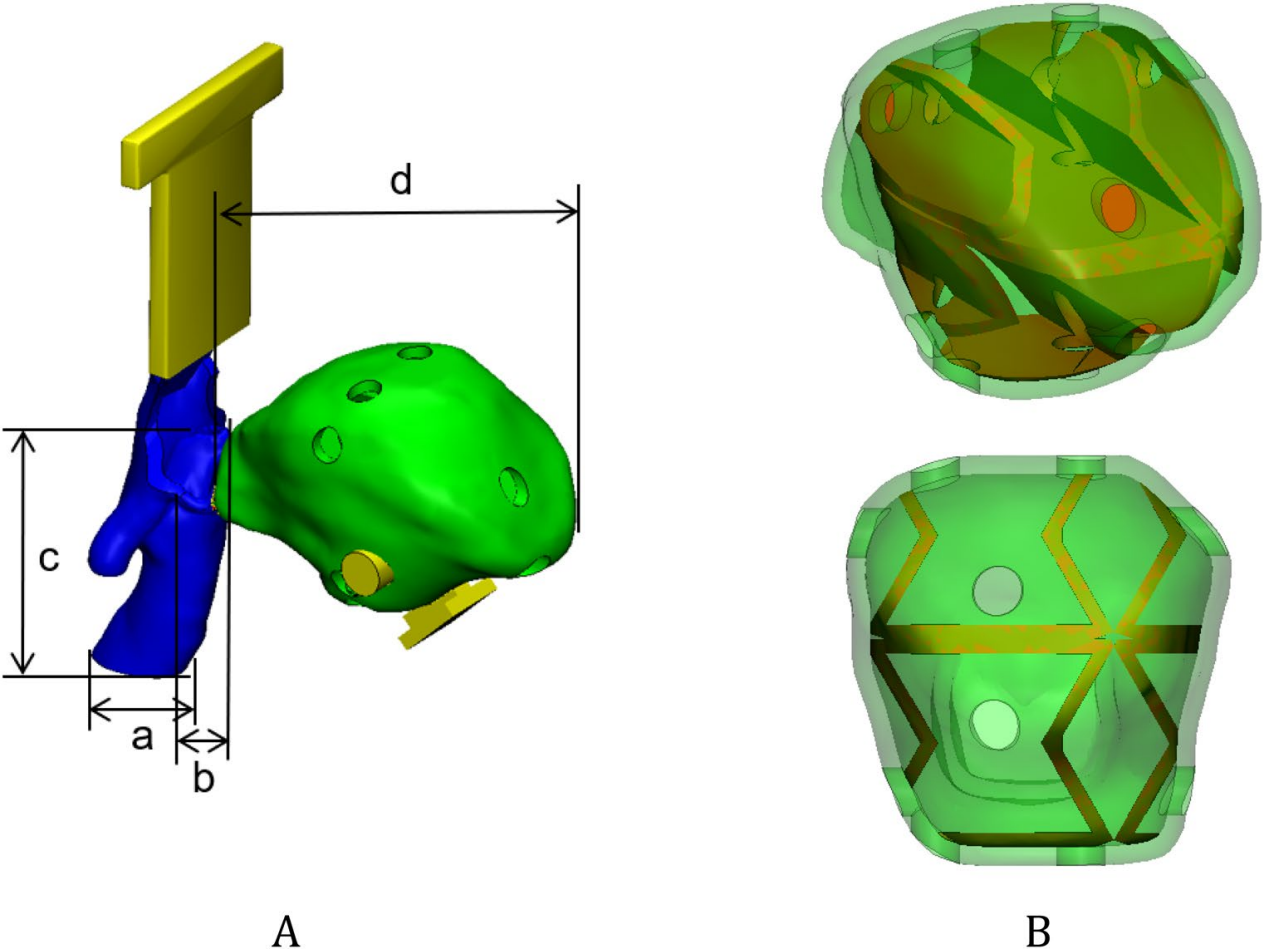
The airway and tongue were modelled based on CT images of a patient with Crouzon syndrome. (**A**) One part with the airway (blue) and tongue (green), and four measurements for evaluating fabrication accuracies, including (a) the inner diameter of the airway, (b) the width of the epiglottis, (c) the length between the epiglottis to the end of airway, and (d) the length of the tongue (connectors to a mandible and cranio-maxilla; yellow). (**B**) inner structure (brown) and the outer hole of the tongue to mimic the tactile sensing to press the tongue with Macintosh blades and holes at the isometric (upper) and front (lower) views.

The airways and tongue are very soft and are connected to each other in CT images; thus, it is important for users to perform realistic intubation. Therefore, in this study, the airways and tongue were modelled in one part. Based on the segmentation in the CT, the airway and tongue were modelled by mimicking the actual anatomy of a patient with Crouzon syndrome and included realistic mechanical properties. The tongue is designed mimicking the feeling of being pressed by Macintosh blades through inserting inner structure and making the outer hole (Fig. 7B). The tongue has three joints attached to a mandible to move together (Fig. 7A).

#### Modelling for mouth movement

**Figure 8:**
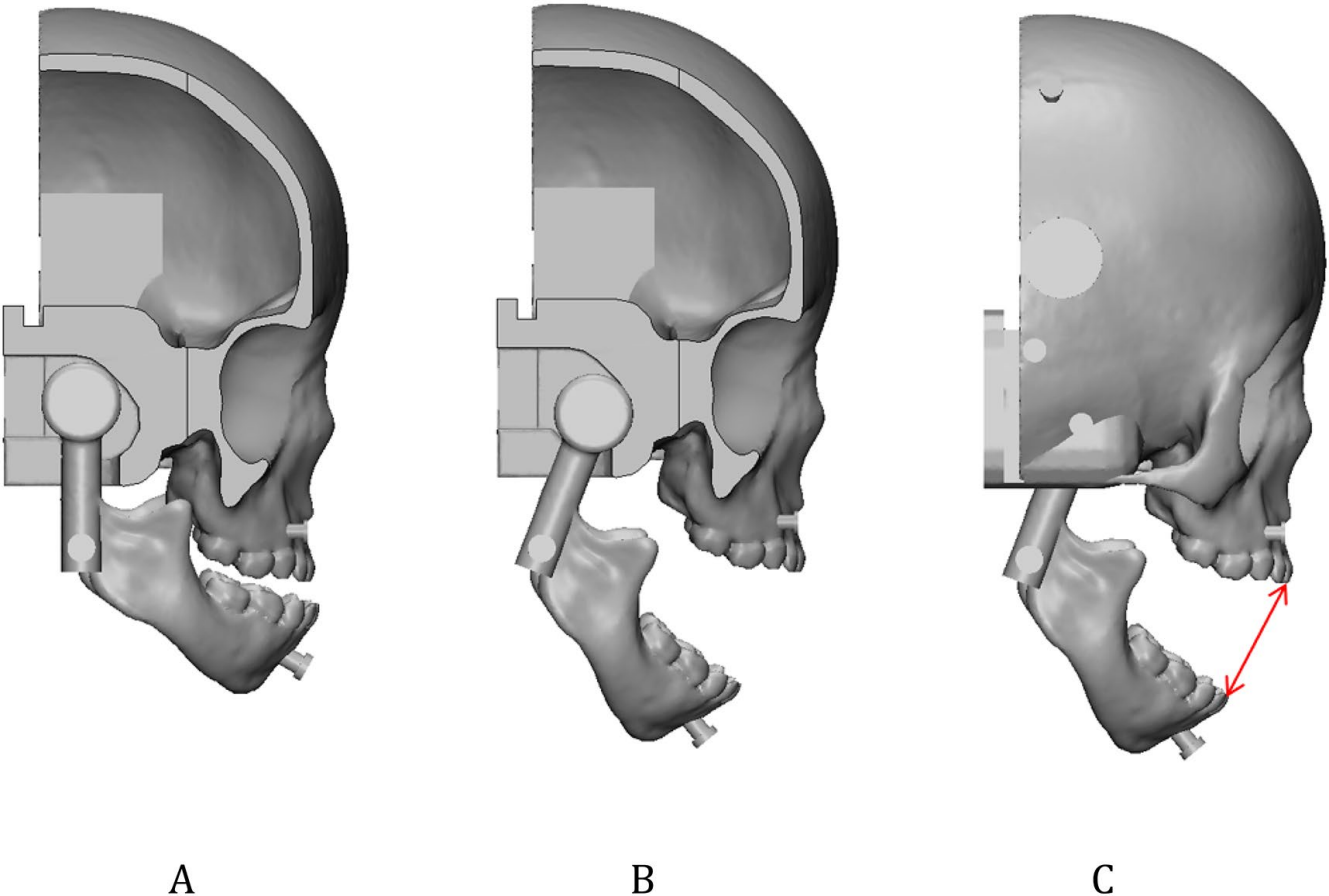
The overall design of the mouth movement. (**A**) The cranio-maxilla with a trailed socket inserted by a circular shaped condyle of the mandible at the closed mouth in the cutting at trailed socket. (**B**) The cranio-maxilla with the slid and rotated mandible at the open mouth in the cutting view. (**C**) At state (**B**), the length of the mouth opening in the inter-incisor distance in 3D visualisation was measured (red line).

The mandible was designed to connect the tongue and the cranio-maxilla parts. The condyle of the mandible was modelled as a circular shape to connect to the cranio-maxilla part. In addition, the cranio-maxilla was connected to the back of the skull and the airway and tongue parts. To mimic realistic mouth movement, the cranio-maxilla part was designed to have a trailed socket for the condyle of the mandible to be inserted and move along (Fig. 8A,B). The trailed socket was designed for the condyle of the mandible to slide 7 mm at a 45° slope and to rotate. The rotation was limited to 11°, 21°, and 35°, and the inter-incisor distance of each angle was 21, 32, and 47 mm in 3D visualisation.

#### C-spine, skull, and skin

**Figure 9:**
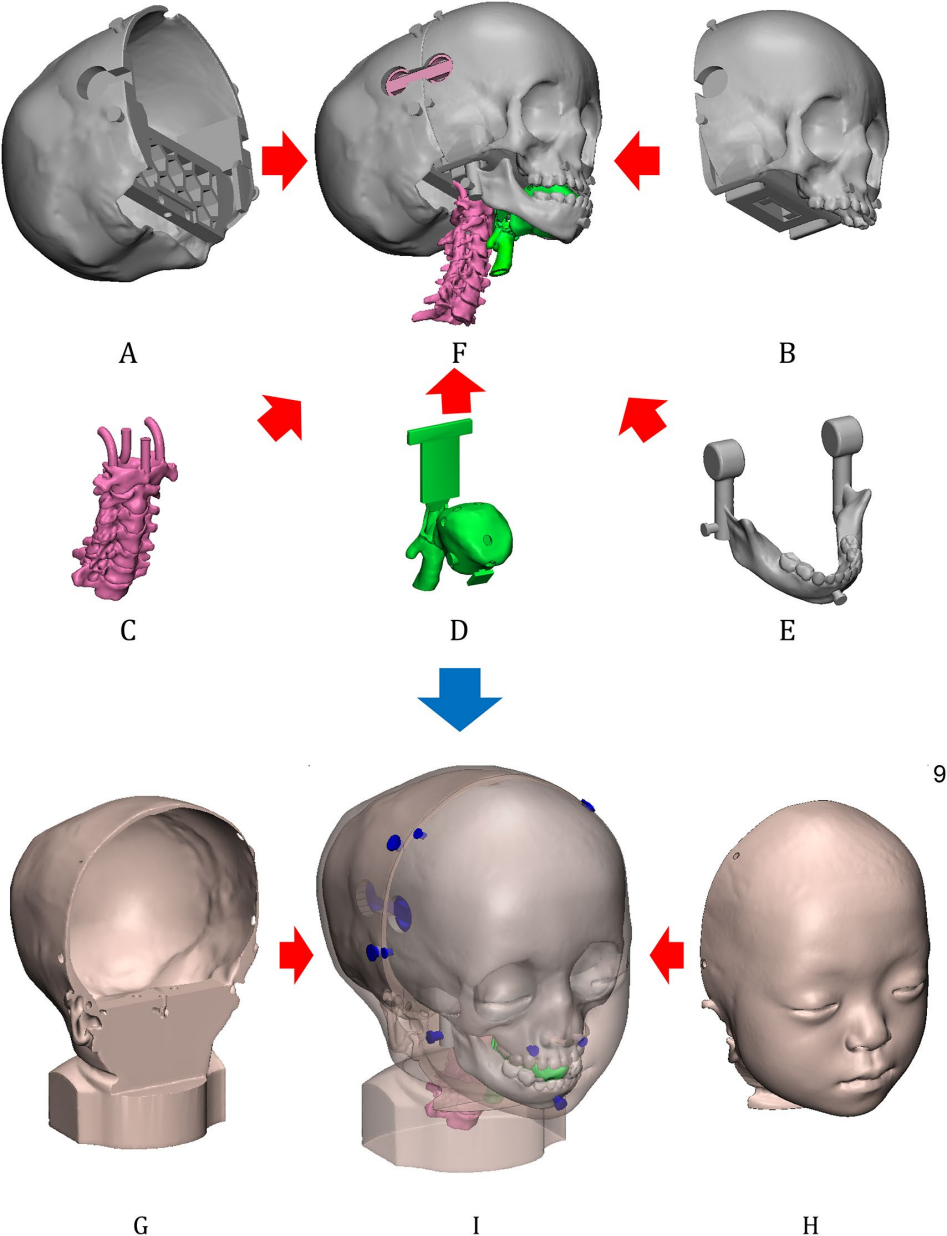
Assembly of a difficult tracheal intubation phantom with various anatomic parts including (**A**) the back of the skull, (**B**) the cranio-maxilla, (**C**) the cervical spine, (**D**) the airway and tongue, (**E**) the mandible, and (**F**) the assembly of the inner structures including (**A**)–(**E**), excluding the skins. With skins including (**G**) rear and (**H**) front parts, all parts are assembled in (**I**) which integrates (**F**)–(**H**). The blue parts of (**I**) were used as connectors of the rear and front skins into the skull.

The c-spine was designed to connect with the back of the skull. It was fabricated of seven separate c-spines, with hooks on the first part for connection with the back of the skull (Fig. 9C). To fabricate the ligaments of the spines, individually fabricated c-spines were inserted into a special moulder for silicone to be poured into the empty spaces to generate ligaments and discs, which mimic natural c-spine movement (Fig. 1A).

The cranio-maxilla and the back of the skull were connected using sliders and connectors. The cranio-maxilla and the mandible were connected using the circular condyles of the mandible and the socket of the cranio-maxilla (Fig. 9F). The skin moulders were designed to manufacture the front and rear parts separately. The front skin moulder was used to create a front skin part, and injection of silicone into the empty space was used to attach the anatomic parts including the cranio-maxilla, mandible, tongue, and the airway (Fig. 9B,D,E). The rear skin moulder was designed to attach to the back of the skull, c-spine (Fig. 9A,C).

### Virtual assembly

Based on the segmentation results, various anatomic parts, including the back of the skull, cranio-maxilla, c-spine, airway, tongue, mandible, and the rear and front skins were modelled using 3-matics v9 (Materialise, Leuven, Belgium). The front anatomy parts are the cranio-maxilla, mandible, airway, and tongue. The rear anatomy parts are the back of the skull and c-spine. After assembling each of the front and rear parts, the inner structures were assembled using the slider and connector between the back of the skull and the cranio-maxilla. Figure 9F depicts the assembly of a difficult tracheal intubation phantom with various inner anatomic parts. Figure 9I represents the assembly of the front and rear skin in Fig. 9F.

### 3D printing and post-processing

A c-spine part consisting of seven spines was independently fabricated using fused deposition modelling (FDM) with thermoplastic polyurethane (TPU), due to their economic price and adequate strength. The c-spine moulder was fabricated using Stereolithography Apparatus (SLA) with clear resin, due to their economic price and ability to check the spread of silicone. The fabricated c-spine part was inserted into the c-spine moulder, and silicone (dragon-skin-10 and eco flex-0010, Smooth-on, USA) were injected into the empty space. The injected dragon-skin-10 and eco flex-0010 have similar material properties to the actual ligaments and discs of c-spines (Fig. 1A). The airways and tongue were fabricated by PolyJet with agilus (Objet J750, Stratasys, USA), SLA with elastic (Form2, Formlabs, USA), and another SLA with flexa 693 (X-fab, DWS Lab, Italy), respectively. In addition, coating with silicone (MED6-6606, NuSil Technology, USA) was performed to achieve a variety of mechanical properties relating to elongation and strength. The skin moulder was manufactured using colour-jet printing (CJP) with plaster (ProJet 460,3D Systems, USA), due to the printing size and considering the removal moulder. After the cranio-maxilla, mandible, and airway parts were inserted into the front skin moulder and the c-spine and the back of the skull were inserted into the rear skin moulder, the moulders were sealed and silicone (Dragon-skin-fx-pro, Smooth-on, USA) was injected into the moulders. The skin moulders were broken and removed after 1 day to allow for sufficient curing of the silicones.

### Evaluation of phantoms

#### Shape accuracies

Three airway and tongue parts of the same STL file were fabricated using two SLA machines and PolyJet, respectively. Bland–Altman analysis was used to evaluate the shape accuracy between the STL file and the three fabricated airway and tongue parts. Two researchers evaluated four lengths of the STL models and 3D printed phantoms 5 times using 3-Matics (Materialise, Belgium) and Vernier Callipers, respectively.

#### Hardness of airway and tongue

Airway and tongue parts were fabricated to have a similar Shore A hardness to the previous study^9^; these parts were fabricated by Objet J750 with agilus, Form2 with elastic, and X-fab with flexa 693, respectively. In addition, these parts were coated 1, 5, and 10 times using MED6-6606. A box plot was used to display the hardness of each part. Three specimens per each condition at the edge and centre were measured five times using a Shore A durometer (Landtek, Guangzhou, China).

#### Accuracy of mouth opening

The inter-incisor distances between at 11°, 21°, and 35° of mouth opening were measured to evaluate the accuracy of mouth opening in the phantom. The Bland–Altman plot was used to evaluate the accuracy of the mouth opening between the STL file and 3D printed models. Two researchers independently measured each inter-incisor distance of the STL models and 3D printed phantoms 5 times using 3-Matics and Vernier callipers, respectively.

### Ethical statement

The study was approved by the Institutional Review Board of Asan Medical Center (IRB no. 2018-0967) and was performed in accordance with the principles of the Declaration of Helsinki. Participants' parents signed a consent form stating voluntary consent for all procedures related to the study and anonymously received a CT image of a toddler patient with Crouzon syndrome with a 1.25 mm slice thickness. Also, a Statement on written informed consent from the parents is provided in the supplementary information.
